# Disulfiram use is associated with lower risk of COVID-19: A retrospective cohort study

**DOI:** 10.1371/journal.pone.0259061

**Published:** 2021-10-28

**Authors:** Nathanael Fillmore, Steven Bell, Ciyue Shen, Vinh Nguyen, Jennifer La, Maureen Dubreuil, Judith Strymish, Mary Brophy, Gautam Mehta, Hao Wu, Judy Lieberman, Nhan Do, Chris Sander

**Affiliations:** 1 Boston VA Cooperative Studies Program (CSP) Center, VA Boston Healthcare System, Boston, Massachusetts, United States of America; 2 Department of Medicine, Harvard Medical School, Boston, Massachusetts, United States of America; 3 Department of Clinical Neurosciences, University of Cambridge, Cambridge, United Kingdom; 4 Department of Cell Biology, Harvard Medical School, Boston, Massachusetts, United States of America; 5 Department of Data Science, Dana-Farber Cancer Institute, Boston, Massachusetts, United States of America; 6 Broad Institute of Harvard and MIT, Boston, Massachusetts, United States of America; 7 Section of Rheumatology, Boston University School of Medicine, Boston, Massachusetts, United States of America; 8 Rheumatology, VA Boston Healthcare System, Boston, Massachusetts, United States of America; 9 Infection Disease, VA Boston Healthcare System, Boston, Massachusetts, United States of America; 10 Section of Hematology and Medical Oncology, Boston University School of Medicine, Boston, Massachusetts, United States of America; 11 Institute for Liver and Digestive Health, University College London, London, United Kingdom; 12 Institute of Hepatology, Foundation for Liver Research, London, United Kingdom; 13 Department of Biological Chemistry and Molecular Pharmacology, Harvard Medical School, Boston, Massachusetts, United States of America; 14 Program in Cellular and Molecular Medicine, Boston Children’s Hospital, Boston, Massachusetts, United States of America; 15 Department of Pediatrics, Harvard Medical School, Boston, Massachusetts, United States of America; 16 Section of General Internal Medicine, Boston University School of Medicine, Boston, Massachusetts, United States of America; University of Hail, SAUDI ARABIA

## Abstract

Effective, low-cost therapeutics are needed to prevent and treat COVID-19. Severe COVID-19 disease is linked to excessive inflammation. Disulfiram is an approved oral drug used to treat alcohol use disorder that is a potent anti-inflammatory agent and an inhibitor of the viral proteases. We investigated the potential effects of disulfiram on SARS-CoV-2 infection and disease severity in an observational study using a large database of clinical records from the national US Veterans Affairs healthcare system. A multivariable Cox regression adjusted for demographic information and diagnosis of alcohol use disorder revealed a reduced risk of SARS-CoV-2 infection with disulfiram use at a hazard ratio of 0.66 (34% lower risk, 95% confidence interval 24–43%). There were no COVID-19 related deaths among the 188 SARS-CoV-2 positive patients treated with disulfiram, in contrast to 5–6 statistically expected deaths based on the untreated population (P = 0.03). Our epidemiological results suggest that disulfiram may contribute to the reduced incidence and severity of COVID-19. These results support carefully planned clinical trials to assess the potential therapeutic effects of disulfiram in COVID-19.

## Introduction

As of September 2021, there have been more than 210 million cases of coronavirus disease 2019 (COVID-19) worldwide with over 4.5 million deaths. Although several vaccines against severe acute respiratory syndrome coronavirus 2 (SARS-CoV-2) have been approved and are being administered internationally, there will still be a significant number of infections in people who are not vaccinated or for whom vaccination fails to fully protect, especially in regions with inadequate access or acceptance of vaccination. Moreover, there is a growing need for effective treatment of COVID-19, especially in light of reduced vaccine effectiveness in preventing infection with novel mutant viruses. Thus, identifying effective, inexpensive, and widely accessible treatment strategies is a high priority.

Repurposing of medications that are already approved for other indications is an attractive strategy, given the length of time and costs involved in developing new drugs [1]. With a growing number of candidates being identified, conducting a clinical trial for each remains a challenge due to the substantial time and financial resources required. While not equivalent to a randomized controlled study, analysis of real world clinical records can be used to investigate the incidence or severity of disease in those prescribed a medication compared to those not on the drug and provide observational evidence of a drug’s potential effectiveness against COVID-19https://paperpile.com/c/94FeSo/ysdv [1]. This study focuses on disulfiram (Antabuse), which has been used for over 60 years to deter alcohol use as it inhibits aldehyde dehydrogenase and causes alcohol flush reaction [2]. Disulfiram potentially targets multiple enzymes because it is a Cys-reactive drug. Despite its lack of specificity, it has an exceedingly favorable safety profile in the absence of concomitant alcohol use. The study was motivated both by promising observations about the effects of disulfiram in biochemical and cell-based screens and in mouse experiments [3–12].

There are several indications regarding the potential effects of disulfiram in reducing infections with SARS-CoV-2 and severity of COVID-19, as follows: (i) Inhibition of the main protease: Jin *et al*. [3] conducted high throughput screening of potential inhibitors of the SARS-CoV-2 main protease (M^pro^), an enzyme needed for viral replication and identified disulfiram (IC_50_ = 9.35 μM) among the candidates. (ii) Inhibition of the papain-like protease: earlier Lin *et al*. [4] demonstrated the inhibition of the papain-like proteases (PL^pro^) of MERS-CoV and SARS-CoV-1 by disulfiram through multiple enzymatic assays. The inhibition of PL^pro^ of SARS-CoV-2 has been confirmed by several groups, at IC_50_ values in biochemical assays ranging from 2–7 μM [5–7]. (iii) Inhibition of other viral proteins: disulfiram has been reported to inhibit the ATPase activity of Nsp13 and the exoribonuclease activity of Nsp14 of SARS-CoV-2 via its Zn^2+^-ejector function [11]. (iv) In addition, disulfiram has general anti-inflammatory effects in sepsis. Based on the results of a drug screen of 3,752 compounds, Hu *et al*. [12] discovered a pronounced anti-inflammatory effect of disulfiram that reduced mortality in a mouse model of sepsis. Disulfiram inhibits inflammatory cytokine IL-1β release and pyroptosis (inflammatory cell death) by blocking the assembly of the gasdermin D pore by covalent modification of Cys191. These mechanistic studies support the hypothesis that disulfiram may be a polypharmacological therapeutic compound preventing both virus replication and life-threatening host response in coronavirus disease.

## Materials and methods

### Data and patients

The data used for this retrospective cohort study comparing Veterans who received treatment with disulfiram to those who were not treated, is from the Department of Veterans Affairs (VA) COVID-19 Shared Data Resource and the VA Corporate Data Warehouse (CDW), which consolidates electronic health record data from VA facilities nationwide. We identified a cohort of Veterans with at least one SARS-CoV-2 laboratory test result between February 20, 2020 and February 1, 2021. The tests include any test done at a VA facility, which encompasses both hospital inpatient and outpatient visits as well as primary care. In addition, we required that each Veteran had visited a VA primary care provider in the 18 months before their first SARS-CoV-2 test. Within this cohort, we identified 2,233 Veterans (median age 51 years [IQR 39, 61], 8.4% female) with at least one pharmacy record for disulfiram between February 20, 2019 and February 1, 2021, and 941,894 Veterans (median age 64 years [IQR 51, 72], 11.6% female) not prescribed disulfiram between those dates. Disulfiram pharmacy records were identified in the CDW based on standardized generic drug names. This study was approved as an exempt study by the VA Boston Healthcare System Research and Development Committee. The data used for this study were identifiable and the requirement for informed consent was waived.

### Study variables and outcomes

The primary outcome of interest was the occurrence of a positive test result for SARS-CoV-2 (either PCR or antigen), as recorded in the VA COVID-19 Shared Data Resource. Patients were followed from February 20, 2020 through the date of a positive SARS-CoV-2 test, the date of death, or the end of the study (February 1, 2021), whichever came first. Date of death was defined based on mortality records available in the CDW, integrating data from Medicare, Social Security Administration, VA facilities, National Cemetery Administration, and death certificates [13]. In addition to the primary outcome, we also considered severe clinical outcomes subsequent to SARS-CoV-2 infection, including death, intensive care unit (ICU) admission, and mechanical ventilation; only events occurring within 2 weeks of a positive SARS-CoV-2 test were considered. Details of these outcome definitions are described in our previous work [14]. Note, cause of death as recorded on a death certificate is not used to determine whether a death was COVID-19-related, as this may be incomplete or inaccurate. For a composite severe endpoint, we combined death, ICU admission, and mechanical ventilation.

Study variables included age at start of follow-up (i.e., February 20, 2020), gender, race/ethnicity, region of the VA facility where each patient was tested for SARS-CoV-2, diagnosis of alcohol use disorder (AUD), and the Charlson comorbidity index. Age, gender, race, and ethnicity were defined based on structured data in the CDW. The region of the VA facility where each patient was tested for SARS-CoV-2 was determined using a mapping previously reported [14]. Diagnosis of AUD in the year prior to the start of follow-up was determined based on an algorithm adapted for VA from the Centers for Medicare and Medicaid Chronic Conditions Warehouse algorithms [15]. The Charlson comorbidity index was calculated using the R *comorbidity* package based on ICD-10 codes recorded in the year prior to the start of follow-up [16]. Receipt of at least one dose of a SARS-CoV-2 vaccine during the study period (i.e., on or prior to February 1, 2021) was determined using the VA COVID-19 Shared Data Resource.

### Statistical analysis

We summarized patients’ demographics at baseline, i.e., measured at or before the start of follow-up, for the full analytic sample and by disulfiram treatment status. We also tested for baseline differences by treatment status using t-tests for continuous variables and chi-squared tests for categorical variables.

We conducted two sets of analyses to examine the associations between disulfiram treatment and SARS-CoV-2 test positivity. First, we calculated the incidence rate of SARS-CoV-2 infection during follow-up time before and after disulfiram treatment, and assessed differences using the incidence rate ratio and a Wald test. Patients were considered to have been treated with disulfiram during all follow-up time on or after the date of their first pharmacy record for a dispensed disulfiram prescription, as in an intention-to-treat analysis. If the patient received disulfiram between February 20, 2019 and February 20, 2020, all follow-up time was coded as post-treatment. If the positive test occurred during follow-up, the follow-up time before the first pharmacy record was coded as pre-treatment and time after that record was coded as post-treatment. For the untreated cohort, if the patient did not have any disulfiram pharmacy records, all follow-up time was coded as pre-treatment.

Second, we fit univariable and multivariable Cox proportional hazards models, adjusting for covariates listed in S1 Table. In multivariable analysis, age was included as a continuous variable, measured in years, while other variables were categorical. While the primary approach assumed that those who were treated with disulfiram continued to be exposed throughout the remaining follow up time, we also performed a sensitivity analysis considering that patients remained on disulfiram for only one month after each record of dispensation. Another sensitivity analysis was restricted to those with diagnosis of AUD. Analyses were conducted using R v4.0.2 (http://www.r-project.org) and P-values < 0.05 were considered statistically significant.

## Results

In this study, we looked for epidemiological evidence to be considered when prioritizing existing medications for clinical trials in both the early and late stages of COVID-19. We investigated the incidence and outcome of COVID-19 disease among persons taking disulfiram compared to the general population. We conducted a retrospective cohort study comparing Veterans who were treated with disulfiram to those who were not treated. The data is from the US Department of Veterans Affairs (VA) COVID-19 Shared Data Resource and the VA Corporate Data Warehouse (CDW), which consolidates electronic health record data from VA facilities nationwide. We identified a cohort of Veterans with at least one SARS-CoV-2 laboratory test result between Feb. 20, 2020 and Feb. 1, 2021. In order to ensure patients were receiving routine care at the VA, we required that each Veteran had visited a VA primary care provider in the 18 months before their first SARS-CoV-2 test.

**Fig 1:**
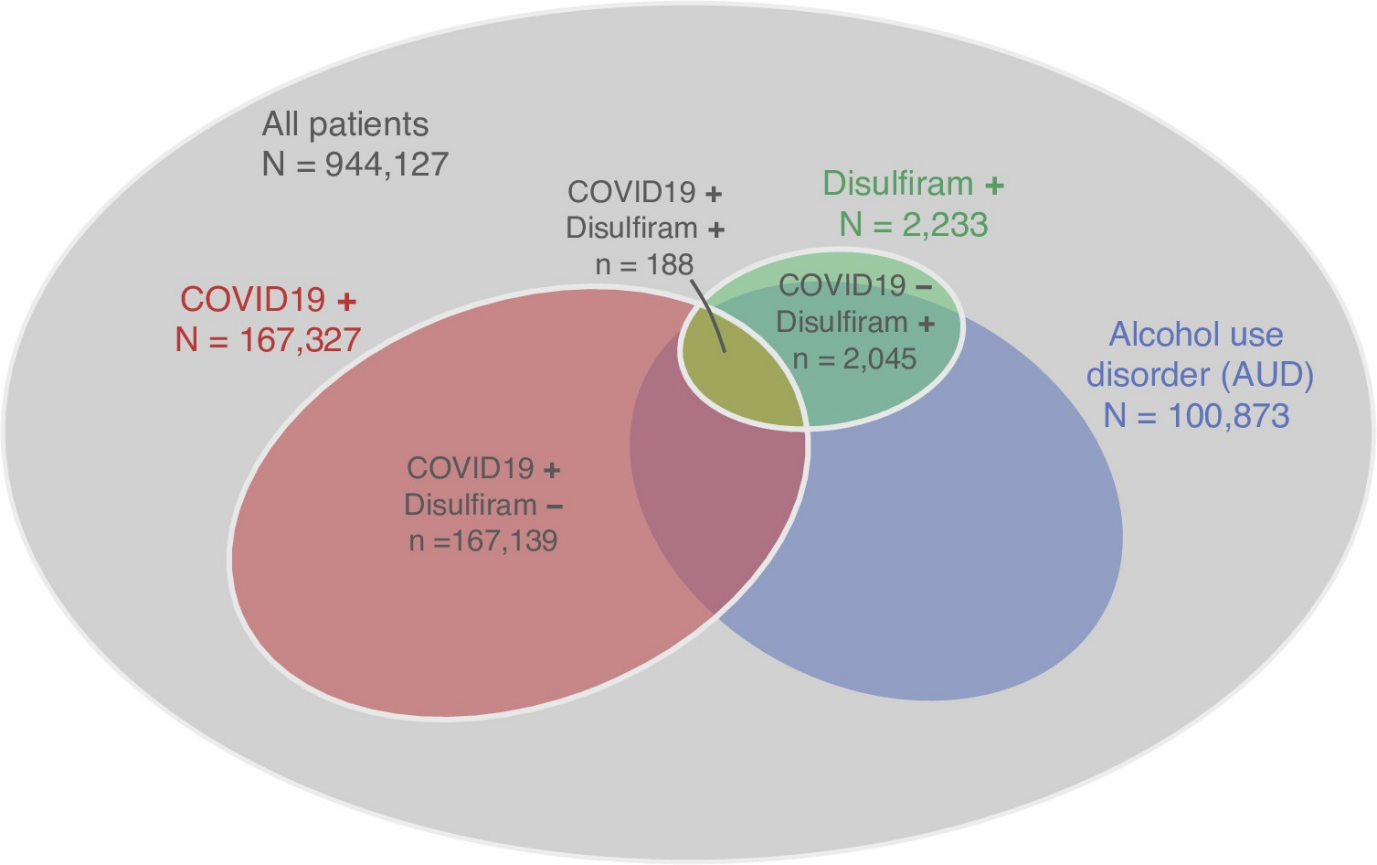
Population distribution of the patient cohort used for the study. 944,127 Veterans have at least one SARS-CoV-2 laboratory test result between Feb. 20, 2020 and Feb. 1, 2021, and had visited a VA primary care provider in the 18 months before their first SARS-CoV-2 test. Disulfiram + (-) indicates at least one (no) pharmacy record for disulfiram on or after Feb. 20, 2019. COVID19 + (-) indicates a positive (no positive) SARS-CoV-2 laboratory test.

Among 944,127 patients meeting the inclusion criteria, 2,233 had at least one pharmacy record for disulfiram on or after Feb. 20, 2019 and 100,873 had been diagnosed with AUD (Fig 1). The patient population is racially and regionally heterogeneous, reflecting the VA’s nationwide patient population (S1 Table). Patients treated with disulfiram differed in their basic demographic and clinical history from those who were not treated, e.g., they were younger, more likely to be white, and having a lower burden of comorbidity (P < 0.001 for all tests). Therefore these potentially confounding variables were taken into account in the regression analysis.

The incidence of SARS-CoV-2 infection in patients treated with disulfiram was 188 patients infected during 1,674 person years of follow-up. In contrast, the incidence of infection in untreated patients was 167,139 patients infected during 838,279 person years of follow-up. Reflecting this difference, the incidence rate ratio of infection in disulfiram-treated vs untreated patients was 0.56 (95% confidence interval (CI): 0.49–0.65, P < 0.001), i.e., a 44% lower rate of infection in those treated with disulfiram.

In a simple univariable Cox regression model, we observed a hazard ratio (HR) of 0.50 (95% CI 0.44–0.58, P < 0.001), similar to the incidence rate ratio, as expected. In the more refined multivariable model (Table 1, unrestricted analysis), after adjusting for age, gender, race/ethnicity, region, Charlson score (a comorbidity index), and AUD diagnosis, the association was still highly significant. Patients treated with disulfiram had a 34% lower risk of SARS-CoV-2 infection compared to those not treated with disulfiram, i.e., an HR of 0.66 (95% CI 0.57–0.76, P < 0.001). The multivariable model also revealed significant associations of risk of infection with age, gender, race/ethnicity, region, and comorbidity (via the Charlson score), which are consistent with earlier results [14].

**Table 1:** Multivariable Cox regression results for a positive SARS-CoV-2 test against disulfiram treatment and other factors.

		Unrestricted analysis Hazard Ratio (95% CIs^a^)	P-value	Restricted analysis Hazard Ratio (95% CIs)	P-value
**Disulfiram**	**Treated**	**0.66 (0.57, 0.76)**	**<0.001**	**0.68 (0.58, 0.80)**	**<0.001**
**AUD** ^**b**^	Diagnosed	0.69 (0.68, 0.70)	<0.001		
**Gender**	Female	1.00 (ref.^c^)			
	Male	1.18 (1.16, 1.20)	<0.001	1.12 (1.05, 1.20)	<0.001
**Age**	Continuous increase	1.00 (1.00, 1.00)	<0.001	0.99 (0.99, 1.00)	<0.001
**Ethnicity**	Non-Hispanic White	1.00 (ref.)			
	Non-Hispanic Black	1.10 (1.09, 1.11)	<0.001	1.17 (1.12, 1.21)	<0.001
	Hispanic	1.33 (1.31, 1.35)	<0.001	1.70 (1.61, 1.79)	<0.001
	Other/Unknown	1.12 (1.10, 1.14)	<0.001	1.19 (1.12, 1.27)	<0.001
**Region**	Continental	1.00 (ref.)			
	Midwest	1.02 (1.00, 1.03)	0.038	1.01 (0.96, 1.06)	0.736
	North Atlantic	0.71 (0.70, 0.72)	<0.001	0.77 (0.73, 0.81)	<0.001
	Pacific	0.65 (0.64, 0.66)	<0.001	0.79 (0.75, 0.84)	<0.001
	Southeast	0.78 (0.77, 0.79)	<0.001	0.78 (0.74, 0.83)	<0.001
**Charlson score** ^**d**^	0	1.00 (ref.)			
	1–2	0.96 (0.95, 0.97)	<0.001	0.91 (0.87, 0.94)	<0.001
	3–4	0.87 (0.85, 0.88)	<0.001	0.89 (0.83, 0.94)	<0.001
	≥5	0.83 (0.81, 0.85)	<0.001	0.92 (0.85, 1.00)	0.046
	Unknown	1.06 (1.03, 1.09)	<0.001	0.95 (0.55, 1.63)	0.844

Unrestricted analysis stands for multivariable Cox regression on all 944,127 patients meeting the inclusion criteria; Restricted analysis was performed on the 100,873 patients diagnosed with AUD.

^a^Confidence intervals

^b^Alcohol Use Disorder

^c^Reference category

^d^Charlson comorbidity index.

To further separate the effect of AUD (HR = 0.69 in the unrestricted analysis) and disulfiram, we performed the multivariable Cox regression analysis restricted to those with diagnosis of AUD (100,873 out of 944,127 patients) (descriptive statistics for the results sample are presented in S2 Table). We observed an HR of 0.68 (95% CI 0.58–0.80, P < 0.001) (Table 1, restricted analysis). Within confidence limits, this result is consistent with the HR of 0.66 of the unrestricted analysis.

In the main analysis, motivated by potential incompleteness of data capture and the relatively short length of followup (<1 year maximum) in our study, we assume that each patient continues disulfiram treatment starting from their first disulfiram pharmacy record through the end of their followup. As an additional sensitivity analysis, we fit a multivariable model with the same covariates but assumed that patients remained on disulfiram for only one month after each record of dispensation, and observed a similar association as in the primary analysis (HR = 0.53; 95% CI 0.44–0.64, P < 0.001).

Finally, it is possible that our results could be affected by SARS-CoV-2 vaccination, which began during the study period. As a third sensitivity analysis, we fit a multivariable model with the same covariates but with followup time censored on December 11, 2020, which is the date of the first dispensation of a SARS-CoV-2 vaccine at the VA, and we again observed a similar association (HR = 0.64; 95% CI 0.55–0.74, P < 0.001). Of note, patients treated with disulfiram were less likely to be vaccinated against SARS-CoV-2 during our study period (11.6%) compared to those untreated (15.7%; P < 0.001; S1 Table), which if anything would result in an underestimation of the HR for those on disulfiram.

We also analyzed whether disulfiram prescription was linked to severe clinical outcomes and a composite measure of severe outcomes (Table 2). Inferences from these data are limited due to the low frequency of events. There were no statistically significant differences in ICU admissions and mechanical ventilation between the two cohorts. However, we did observe a statistically significant difference in occurrence of COVID-19 related death. In the untreated cohort, 3% of patients experienced COVID-19 related death. In contrast, among the 188 patients treated with disulfiram, no COVID-19 related deaths were reported at all (P = 0.03, Table 2), contrasting with 5–6 that would have been expected under a 3% incidence proportion. Nevertheless, overall, there was no statistically significant difference between the proportion of the composite severe endpoints between the two cohorts. Given the low counts of severe clinical outcomes in this study and uncertainty regarding drug adherence and potential discontinuation on admission, epidemiological observations in a larger dataset or in an inpatient setting are needed to probe whether disulfiram has an impact on the severity of COVID-19.

**Table 2:** Clinical outcomes in patients infected with SARS-CoV-2 by disulfiram treatment status.

	No. of treated patients (N = 188)	No. of untreated patients (N = 167,139)	Incidence proportion in treated	Incidence proportion in untreated	P-value
**ICU** ^**a**^	11	7,403	0.059	0.044	0.44
**Mechanical ventilation**	1	959	0.005	0.006	1.00
**Death**	**0**	**5,009**	**0.000**	**0.030**	**0.03**
**All severe outcomes**	12	13,371	0.063	0.080	0.45

^a^Intensive care unit.

## Discussion

Our study is the largest using real world data to demonstrate a lower risk of SARS-CoV-2 infection in those taking disulfiram. Recently, in a preliminary report, Tamburin *et al*. [17] showed that patients taking disulfiram had a significantly lower incidence of symptoms compatible with COVID-19, such as fever and dyspnea, compared to a control group. However, their study was underpowered to discern a difference in actual risk of COVID-19 disease. Our study significantly extends these initial observations in two ways: (1) the VA patient population with relevant data is considerably larger, which enabled us to infer a statistically significant association between disulfiram use and risk of SARS-CoV-2 infection, and (2) we were able to document the clinical outcomes of those infected with COVID-19 taking disulfiram and estimate the risk of death. Other commonly used drugs have also been reported to reduce the risk of infection and disease severity [18]. Access to larger clinical datasets and meta-analysis across diverse health-care systems would be useful in future investigations on this topic.

The principal limitation of our study, as is true even for studies with controlled cohorts, is that association does not prove causation and that even very likely causation does not guarantee the success of the corresponding therapeutic intervention. There are several additional limitations. Since Veterans can receive care outside of the VA, diagnoses and outcomes may be incompletely recorded and we do not know if patients actually took the prescribed disulfiram drug, or whether they were still taking the drug near the time of infection. We also cannot rule out the possibility that those prescribed disulfiram had other factors, such as behavioral differences or other confounding variables, that contributed to their protection against COVID-19. Additionally, we are unable to distinguish symptomatic from asymptomatic infection, as documentation of the relevant symptoms is inconsistent in the electronic health records used in our study. For inpatient settings, patients are routinely tested on admission. For outpatient settings, testing can occur for symptoms or concerns of exposure.

Our epidemiological results suggest that disulfiram may be efficacious in combating COVID-19. Additional information is expected from two small ongoing Phase II clinical trials of disulfiram involving early mild-to-moderate symptomatic (NCT04485130, 60 participants) or hospitalized (NCT04594343, 200 participants) COVID-19 patients. Reduction in disease progression in the first trial, if observed, may be primarily due to inhibition of viral replication. This hypothesis is based on clear evidence from biochemical experiments that disulfiram inhibits several enzymes involved in viral replication (M^pro^, PL^pro^, Nsp13 and Nsp14) via covalent modification of cysteine residues in the active site of the M^pro^ and PL^pro^ proteases and via weakening of Cys-coordinated Zn^2+^ ion binding sites in the finger domain of PL^pro^ and in the RNA-modifying enzymes Nsp13 and Nsp14 [3–11]. Better outcomes in the second trial, if observed, may be more influenced by the anti-inflammatory effects of disulfiram, as demonstrated in a mouse model of sepsis, via inhibition of the formation of the host gasdermin D pore and reduction of pyroptosis and inflammatory cytokine secretion [12].

Given the urgent medical need, larger clinical trials stratified for early and late COVID-19 disease as well as in uninfected populations are highly desirable to test the impact of disulfiram across the entire COVID-19 continuum, from infection to disease progression and severe disease. A positive drug effect would most likely also extend to the constantly evolving SARS-CoV-2 variants and related coronaviruses, as the known viral target sites of disulfiram are highly conserved Cys residues. As a repurposing candidate, disulfiram has particular advantages given its low cost, both in production and distribution, and favorable safety profile [2, 19].

We therefore propose that clinical trials should be pursued proactively, given the current limited access to vaccination and the lack of generally available, low-cost proven therapeutic agents against this pandemic disease. Our view is that such trials require governmental or philanthropic funding and that time is of the essence. If successful in clinical trials, disulfiram would be a good candidate as a COVID-19 therapeutic for world-wide distribution, including to low-income populations.

## Supporting information

S1 Table Patient characteristics in the full analytic sample. The unrestricted analysis was performed on the 944,127 patients with at least one SARS-CoV-2 laboratory, stratified by patients who did and did not receive disulfiram in the study period. Notation for number of patients: N (%); IQR = Interquartile range.
(DOCX)

S2 Table Patient characteristics in the restricted analysis. The restriction is to the 100,873 patients who have a diagnosis of AUD, stratified by patients who did and did not receive disulfiram in the study period. Notation for number of patients: N (%); IQR = Interquartile range.
(DOCX)
