# Supplementary material for: Disulfiram use is associated with lower risk of COVID-19: A retrospective cohort study
Source: PLoS One. 2021 Oct 28;16(10):e0259061. doi: 10.1371/journal.pone.0259061 (PMC8553043; doi:10.1371/journal.pone.0259061)
Supplement: S1 Table — The unrestricted analysis was performed on the 944,127 patients with at least one SARS-CoV-2 laboratory, stratified by patients who did and did not receive disulfiram in the study period. Notation for number of patients: N (%); IQR = Interquartile range. (DOCX) [file pone.0259061.s001.docx]

**S1 Table. Patient characteristics in the full analytic sample.** The unrestricted analysis was performed on the 944,127 patients with at least one SARS-CoV-2 laboratory, stratified by patients who did and did not receive disulfiram in the study period. Notation for number of patients: N (%); IQR = Interquartile range.

| **Patient Characteristics** | **Full Analytic Sample** | **Never Treated with Disulfiram** | **Treated with Disulfiram** |
| --- | --- | --- | --- |
| **All patients** | 944,127 | 941,894 (99.8) | 2,233 (0.2) |
| **Age (median [IQR])** | 64 [51, 72] | 64 [51, 72] | 51 [39, 61] |
| **Gender (%)** |  |  |  |
| Male | 834,982 (88.4) | 832,936 (88.4) | 2,046 (91.6) |
| Female | 109,144 (11.6) | 108,957 (11.6) | 187 (8.4) |
| **Race/Ethnicity (%)** |  |  |  |
| Non-Hispanic White | 573,612 (60.8) | 571,928 (60.7) | 1,684 (75.4) |
| Non-Hispanic Black | 204,379 (21.6) | 204,130 (21.7) | 249 (11.2) |
| Hispanic | 80,154 (8.5) | 80,025 (8.5) | 129 (5.8) |
| Other or Unknown | 85,982 (9.1) | 85,811 (9.1) | 171 (7.7) |
| **Region (%)** |  |  |  |
| Continental | 154,710 (16.4) | 154,449 (16.4) | 261 (11.7) |
| Midwest | 197,872 (21.0) | 197,216 (20.9) | 656 (29.4) |
| North Atlantic | 213,784 (22.6) | 213,214 (22.6) | 570 (25.5) |
| Pacific | 178,755 (18.9) | 178,310 (18.9) | 445 (19.9) |
| Southeast | 199,004 (21.1) | 198,703 (21.1) | 301 (13.5) |
| **Charlson Score (%)** |  |  |  |
| 0 | 361,778 (38.3) | 360,708 (38.3) | 1,070 (47.9) |
| 1-2 | 373,407 (39.6) | 372,503 (39.5) | 904 (40.5) |
| 3-4 | 132,843 (14.1) | 132,657 (14.1) | 186 (8.3) |
| >=5 | 50,689 (5.4) | 50,644 (5.4) | 45 (2.0) |
| Unknown | 25,410 (2.7) | 25,382 (2.7) | 28 (1.3) |
| **History of AUD (%)** | 100,873 (10.7) | 98,910 (10.5) | 1,963 (87.9) |
| **Positive Covid-19 test (%)** | 167,327 (17.7) | 167,139 (17.7) | 188 (8.4) |
| **Received ≥1 Covid-19 Vaccine Dose (%)** | 147,889 (15.7) | 147,637 (15.7) | 252 (11.6) |
