# Supplementary material for: Disulfiram use is associated with lower risk of COVID-19: A retrospective cohort study
Source: PLoS One. 2021 Oct 28;16(10):e0259061. doi: 10.1371/journal.pone.0259061 (PMC8553043; doi:10.1371/journal.pone.0259061)
Supplement: S2 Table — The restriction is to the 100,873 patients who have a diagnosis of AUD, stratified by patients who did and did not receive disulfiram in the study period. Notation for number of patients: N (%); IQR = Interquartile range. (DOCX) [file pone.0259061.s002.docx]

**S2 Table.** **Patient characteristics in the restricted analysis.** The restriction is to the 100873 patients who have a diagnosis of AUD, stratified by patients who did and did not receive disulfiram in the study period. Notation for number of patients: N (%); IQR = Interquartile range.

| **Patient Characteristics** | **Full Analytic Sample** | **Never Treated with Disulfiram** | **Treated with Disulfiram** |
| --- | --- | --- | --- |
| **All patients** | 100,873 | 98,910 | 1,963 |
| **Age (median [IQR])** | 60 [47, 68] | 60 [48, 68] | 51 [39, 60] |
| **Gender (%)** |  |  |  |
| Male | 93,631 (92.8) | 91,813 (92.8) | 1,818 (92.6) |
| Female | 7,242 (7.2) | 7,097 (7.2) | 145 (7.4) |
| **Race/Ethnicity (%)** |  |  |  |
| Non-Hispanic White | 56,366 (55.9) | 54,888 (55.5) | 1,478 (75.3) |
| Non-Hispanic Black | 28,022 (27.8) | 27,804 (28.1) | 218 (11.1) |
| Hispanic | 8,897 (8.8) | 8,782 (8.9) | 115 (5.9) |
| Other or Unknown | 7,588 (7.5) | 7,436 (7.5) | 152 (7.7) |
| **Region (%)** |  |  |  |
| Continental | 16,142 (16.0) | 15,921 (16.1) | 221 (11.3) |
| Midwest | 21,896 (21.7) | 21,322 (21.6) | 574 (29.2) |
| North Atlantic | 23,630 (23.4) | 23,129 (23.4) | 501 (25.5) |
| Pacific | 18,034 (17.9) | 17,637 (17.8) | 397 (20.2) |
| Southeast | 21,171 (21.0) | 20,901 (21.1) | 270 (13.8) |
| **Charlson Score (%)** |  |  |  |
| 0 | 38,724 (38.4) | 37,799 (38.2) | 925 (47.1) |
| 1-2 | 40,641 (40.3) | 39,816 (40.3) | 825 (42.0) |
| 3-4 | 15,002 (14.9) | 14,831 (15.0) | 171 (8.7) |
| >=5 | 6,413 (6.4) | 6,372 (6.4) | 41 (2.1) |
| Unknown | 93 (0.1) | 92 (0.1) | 1 (0.1) |
| **Positive Covid-19 test (%)** | 13,189 (13.1) | 13,025 (13.2) | 164 (8.4) |
| **Received ≥1 Covid-19 Vaccine Dose (%)** | 12,737 (12.6) | 12,518 (12.7) | 219 (11.4) |
